# Punishing Atrocity Crimes in Transitional Contexts: Advancing Discussions on Adequacy of Alternative Criminal Sanctions Using the Case of Colombia

**DOI:** 10.1093/ojls/gqac022

**Published:** 2022-10-11

**Authors:** Beatriz E Mayans-Hermida, Barbora Holá

**Affiliations:** Vrije Universiteit Amsterdam and the Netherlands Institute for the Study of Crime and Law Enforcement (NSCR); NSCR and Associate Professor at Vrije Universiteit Amsterdam

**Keywords:** transitional justice, punishment, alternative sanctions, expressivism, proportionality, restorative justice

## Abstract

Criminal trials and proportional prison sentences are generally seen as the most suitable way to deal with perpetrators of atrocity crimes. Notwithstanding, traditionally conceived criminal penalties, such as imprisonment, may discourage active responsibility-taking by offenders, disaffect victims by not meeting their needs and impede meaningful engagement between perpetrators and survivors. Arguably, alternative criminal sanctions may be appropriate punishment even for atrocity crimes when tried in transitional societies. Using the case of Colombia, this article analyses the justifications of punishment for atrocities in transitional contexts and discusses the adequacy of alternative criminal sanctions as penalties for atrocity crimes. It concludes that under certain conditions, alternative sanctions can be a viable punishment option that may promote active responsibility-taking and contribute to repairing harm, reintegrating offenders into the community and (re)constructing relationships while serving expressive rationales.

## 1. Introduction

After four years of intense negotiations, the Colombian Government and the Fuerzas Armadas Revolucionarias de Colombia-Ejército del Pueblo (FARC-EP)—the biggest guerrilla group in the country—signed the 2016 Final Peace Agreement to End the Conflict and Establish a Stable and Long-lasting Peace (FPA, the Agreement). This agreement is one of several previous attempts to end a five-decade-long internal armed conflict, involving numerous armed state and non-state actors^1^ and resulting in millions of victims.^2^ In contrast to the earlier attempts, the FPA has been considered by the international community as ‘an inspiration for all those striving to end deadly conflict around the world through negotiations’.^3^ Interestingly, the FPA provides a less punitive sanctioning scheme than the already controversial transitional justice (TJ) system established in the so-called Peace and Justice Law of 2005, which provided reduced prison sentences to demobilised paramilitary members.^4^

Based on a *restorative* approach, the FPA envisages the imposition of custodial and non-custodial sanctions for those responsible for the gravest crimes committed within the conflict, including war crimes, crimes against humanity and genocide.^5^ The non-custodial sanctions, also known as *special sanctions* (*sanciones propias*), consist of restriction of rights and liberty in specified geographical areas, coupled with other activities, work and actions with ‘reparative-restorative’ content.^6^ Even the most responsible individuals for the gravest crimes will receive such alternative sanctions (AS) as long as they comply with the conditions established in the Agreement.

Indeed, this unique sanctioning regime has ambitious goals and faces many challenges. The first one is the opposition of some stakeholders within and outside Colombia, who question its leniency and inadequacy as criminal punishment for international crimes.^7^ Non-custodial sanctions or AS^8^ might, at face value, appear inconsistent with the standard on the adequacy of criminal sanctions for international crimes under international criminal law (ICL), international human rights law (IHRL) and international humanitarian law (IHL), which seem to require prison sentences proportional to the gravity of the crime and the culpability of the offender.^9^ However, recent judicial and policy developments at different international courts and tribunals (criminal and human rights) indicate an openness to assessing the adequacy of criminal punishment for international crimes more flexibly, particularly in transitional contexts.^10^

A notable recent example of such increased flexibility of international institutions is the decision of the Office of the Prosecutor (OTP) of the International Criminal Court (ICC) to close, after 17 years, its preliminary examination of the situation in Colombia with a cooperation agreement with the Colombian government signed on the 28 October 2021. In the agreement, the OTP noted Colombia’s ‘demonstrated ability and willingness … to genuinely administer justice related to crimes under the jurisdiction of the [ICC]’ and thus closed the preliminary examination.^11^ For the OTP, therefore, leniency or the non-custodial nature of criminal sanctions alone is not indicative of the inability or unwillingness to bring perpetrators^12^ to justice. However, several questions remain on a theoretical and practical level: can AS designed and delivered in transitional contexts be adequate sanctions for international crimes? What factors should and could be considered when evaluating the adequacy of such sanctions?

According to statements from the former Deputy Prosecutor of the ICC, when assessing reduced and non-custodial sanctions in its complementarity evaluation, the OTP seem to consider the following, largely unspecified, conventional set of factors, amongst others: whether the sanctions serve appropriate sentencing objectives; their proportionality, given the gravity of the crimes and responsibility of the perpetrator; and the type of sanctions.^13^ However, due to the specific character of international crimes and the transitional contexts in which such crimes are being prosecuted and punished, many questions remain: what could ‘the appropriate sentencing objectives’ for punishing international crimes in transitional contexts be? How could AS be justified under such sentencing objectives/punishment rationales? How should the proportionality of such sanctions consequently be assessed? Hence, in this article, using the case study of Colombia, we discuss these challenging questions and offer the first, more or less comprehensive, theoretical framework to assess whether the special sanctions established in the FPA can be seen as adequate for punishing international crimes and why.

The following section will briefly introduce the Colombian 2016 FPA and its sanctioning regime. Section 3 will address the question of what are the ‘appropriate sentencing objectives’ when punishing atrocity crimes in transitional contexts. It argues that (i) communicative and positive preventive theories (hereafter, expressive theories) seem more appropriate than purely retributive and consequentialist rationales to justify punishment of atrocity crimes delivered in transitional contexts and (ii) AS may conform to expressive punishment rationales. However, to be justified as autonomous sanctions for international crimes, AS must not only be grounded in expressive rationales, but must also adopt stronger restorative justice (RJ) values and processes and TJ goals. Therefore, in section 4, we propose a concept of *(re)constructive sanctions*, which reconceptualises AS as adopting stronger RJ and TJ goals. We also further develop what we previously called the *contextualized principle of proportionality*^14^ to assess the adequacy of alternative criminal sentences delivered in transitional contexts. In section 5, we use the case of Colombia—specifically, the sanctioning scheme designed in the FPA—to illustrate the promises (eg punishing crimes, satisfying victims’ rights, resocialisation of offenders), challenges (eg opposition of different stakeholders, practical issues for implementation) and dilemmas (eg incentivise offenders to take active responsibility in exchange of lenient punishment) of this approach. Lastly, section 6 presents the conclusions.

## 2. Colombia’s Final Peace Agreement and Its Sanctioning Regime

The complicated talks in Havana between the FARC-EP and the Colombian government resulted in the adoption of a comprehensive agreement that includes six interconnected points: (i) rural reform; (ii) political participation; (iii) illicit drug policy; (iv) ceasefire, disarmament and demobilisation; (v) victims’ rights; and (vi) implementation and verification, where the United Nations (UN) has a central role.^15^ The point five of the agreement created an Integrated System of Truth, Justice, Reparation and Non-Repetition (Integrated System) to guarantee victims’ rights and includes the Special Jurisdiction for Peace (SJP), an *independent* judicial mechanism in charge of investigating and punishing the crimes committed within the armed conflict. The FPA has been praised, among other reasons, because it ended a five-decade-long conflict and, unlike previous peace accords, adopted a holistic approach to TJ and ‘clearly identified the conditions that needed to be transformed in order for a durable peace to be possible, and even the mechanisms that should be used to ensure enforcement’.^16^ As discussed in section three, adopting such holistic approach and a broader concept of justice is necessary to adequately respond to mass-wrongdoing in transitional contexts.

Nevertheless, the FPA’s sanctioning regime has been largely contested. On the one hand, for signing the FPA, it was essential to address some historic political demands from the guerrilla group and offer penal incentives for their disarmament and demobilisation. The FARC-EP leaders repeatedly stated that ‘nobody negotiates peace in order to go to prison’.^17^ They made it clear that the guerrilla group would not have rendered its arms without penal benefits. However, according to Freeman and Orozco, this was not ‘merely borne of an interest of impunity’; the FARC-EP considered themselves as ‘political offenders’^18^ who did not recognise Colombia’s constitutional order and thus the legitimacy of the state and its institutions. On the other hand, the Colombian government tried to fulfil its international duties to investigate, try and punish international crimes. Hence, the justice item was one of the longest and most challenging to agree upon during the negotiations.^19^

Against this backdrop, the resulting negotiated sanctioning regime sought to strike a balance between the demands of both actors, while guaranteeing victims’ rights. The negotiating parties agreed to provide amnesties and pardons for political offences, such as rebellion, sedition, rioting and conspiracy. For international crimes, the sanctioning regime provides for three types of penal sanctions: (i) special sanctions; (ii) reduced prison sanctions (*sanciones alternativas*); and (iii) ordinary prison sanctions (*sanciones ordinarias*).^20^ In line with the Integrated System, the explicit goals of this regime are to guarantee the satisfaction of victims’ rights and to consolidate peace.^21^

The special sanctions are envisaged as self-standing penal sanctions that consist of restriction of liberty in specified geographical areas, coupled with other activities, work and actions (TOAR, to use its Spanish acronym). Examples of these activities are the construction of schools, roads and health centres; substituting illicit crops; participating in programmes aiming to protect the environment; removing mines and other explosives, etc.^22^ According to the SJP, the TOAR have a reparative-restorative nature and seek to: (i) repair and restore the damage done to victims; (ii) reintegrate the offenders; and (iii) restore the social bonds and transform society to achieve positive peace.^23^

Those ‘most responsible’ for the ‘most serious’ and representative crimes will receive special sanctions if they comply with the conditionality regime (*régimen de condicionalidad*), which requires them to disclose the complete truth, to make efforts to repair the damage caused to victims and to guarantee non-repetition. In the case of the FARC-EP, individuals must also have previously disarmed and demobilised.^24^ Furthermore, perpetrators must disclose the truth and acknowledge their responsibility before the Chamber for the Acceptance of Truth and Responsibility and Establishment of the Facts in a *dialogical/deliberative* process at the beginning of the procedure. Those considered as having a ‘determinative participation’ in the commission of the crimes will receive special sanctions of five to eight years, and those not seen as determinative, between two and five years.^25^ If the accused denies their responsibility and/or does not accept the facts indicated in the Chamber’s resolution, the Trial Section will conduct an adversarial trial. If the accused contributes with the truth and accepts liability before the judgment, they will receive a reduced prison sentence of five to eight years. In case the accused continues to deny the crimes and is convicted, they will receive ordinary sanctions consisting of imprisonment between 15 and 20 years.^26^ Thus, the sanctioning scheme is ‘based on a logic of incentives and threats, which in turn are mediated by conditions’.^27^

Consequently, the FPA introduces a new understanding of criminal accountability and punishment for international crimes, moving away from the traditional conceptualisation of punishment as a moral desert and deterrence to include other modalities and goals, such as repairing the harm done, the resocialisation of the offender and restoring the social tissue affected by the conflict.^28^ Thus, given the unprecedented character of the regime, particularly the nature and modalities of the special sanctions, many questions remain regarding their justifications and adequacy. This is even more so given the predominantly retributive orientation of the Colombian Criminal Code and the fact that Colombia has the duty to investigate and try the perpetrators of the gravest crimes as a party to the Rome Statute of the ICC. Therefore, the Colombian case offers an important context for addressing the theoretical inquiries proposed in this article.

At both the international and national levels, the dominant aims for punishing individuals for committing international crimes are retribution followed by deterrence.^29^However, several scholars have noted the problems and limitations of retributivist and consequentialist goals to punish crimes resulting from collective and structural violence. As discussed in the next section, such limitations become more acute when national courts punish international crimes in transitional scenarios.

## 3. Punishing Atrocity Crimes in Transitional Contexts—What Are the ‘Appropriate Sentencing Objectives’?

Atrocity crimes generally involve many perpetrators, a large number of victims, as well as bystanders, and are usually committed in complex contexts of political unrest, autocratic regimes or very violent societies.^30^ The nature of these crimes and the contexts in which they take place thus differ from common crimes and ordinary times.^31^ Criminological explanations of genocide, crimes against humanity and war crimes note that their large-scale, widespread and/or systematic nature is a product of broader social, political, economic, cultural and/or historical factors that shape the structural contexts in which they take place. Therefore, international crimes must be understood in terms of their connectivity across the macro-, meso- and micro-levels of criminality that ‘shape particular trajectories of conflict and violence’.^32^

On the macro-level, international social structures and dynamics and state organisations play a role in triggering, legitimising and/or tolerating violence.^33^ Furthermore, in most of these contexts, many groups of people become involved or contribute to committing international crimes, which indicates their collective nature: ‘Individuals no longer act as individuals but as representative of the collective.’^34^ Accordingly, on the meso-level, social processes and organisational culture are important. Therefore, this type of wrongdoing usually becomes normalised. On the micro-level, the motives of individuals for participating may be diverse; however, they are often shaped by the social interactions and role expectations of the community or group to which they belong or are exposed. Consequently, although the behaviour may be unlawful, in most cases it is consistent with the societal and environmental norms in a given setting and point in time.^35^

Understanding the nature and contexts where these crimes are committed is essential to developing appropriate responses to such wrongdoing. Punishment and criminal justice systems are usually seen as the most suitable way of dealing with international crimes. Commentators and practitioners—the majority adopting a retributivist approach—often call for harsh punishment, given the seriousness of the crimes.^36^ However, by only prosecuting and punishing perpetrators, many aspects of mass violence are overlooked.^37^ Therefore, criminologists, TJ and other scholars have questioned the suitability of criminal legal responses as the *only or primary* reaction to mass wrongdoing and have called for the design and implementation of broader measures that can better respond to the different aspects and consequences of mass wrongdoing.^38^

TJ aims to address the legacies of large-scale, systematic and/or widespread past abuses and restore the rule of law in post-conflict or post-repressive societies through a comprehensive policy with core elements such as truth, justice, reparations and guarantees of non-recurrence. Although TJ seeks to include different mechanisms, accountability for crimes tends to be promoted across different contexts as a retributive model of formal legal justice.^39^ However, TJ experts call for flexibility in the type and forms of mechanisms to address past violence more effectively in different cultural contexts.^40^ Furthermore, they advocate for a *thicker concept* of justice and *transformative focus*, which seeks to change the post-conflict status quo—that is, the social, economic and political structures and relationships that allow and promote violence.^41^ Under the overarching aim of transformation, many societies in transition usually have other medium- and long-term goals, for example, recognition of victims, promotion of trust, reconciliation, strengthening the rule of law, democratisation and/or achieving sustainable peace.^42^ Achieving such aims requires adopting bottom-up approaches, local civil initiatives and a range of policies and tools not restricted to conventional penal approaches.^43^

Colleen Murphy, for example, holds that, given the particular circumstances of transitional societies, the fundamental concern of TJ is not giving perpetrators their ‘just desert’ but rather the ‘just pursuit of societal transformation’.^44^ The latter implies (i) transforming the structurally unequal relationships among citizens and between citizens and officials; and (ii) treating victims and victimisers in a proper and fitting manner. Treating them fittingly and appropriately requires the responses to past wrongdoing to contribute to moral aims, such as repudiation of wrongdoing, judging perpetrators for their responsibility, contributing to non-­recurrence, acknowledging the status of the victims as rights bearers and members of the political community and providing reparations, among others. For Murphy, the cultural context is also salient for responding to wronging. Many of these moral aims are expressive in nature, and therefore one needs to consider what types of processes could be seen as expressive in specific contexts.^45^

While, in certain cultural contexts, criminal justice and punishment may still have an important role in reinforcing the rule of law, imprisonment becomes undesirable.^46^ Among several reasons, the idea of being imprisoned can even obstruct giving up power to allow a transition or prevent conflicting parties to put an end to a conflict, as in the Colombian case. Therefore, transitional contexts require critical engagement with the dominant retributive focus of punishment and sanctions, and the traditional criminal justice principles and goals to reconceptualise them more flexibly.

### A. Limitations of Retributivism and Deterrence

Purely retributive theories hold that ‘just punishment’ is the one that responds to a crime in a backward-looking way—without any future purpose—and is proportionate to the seriousness of the crime and the offender’s blameworthiness. According to Murphy, retributive punishment is not an appropriate and fitting response to either wrongdoers or victims of international crimes because it inflicts additional wrong through suffering and cannot restore a situation of equality between victims and victimisers, as some retributivists affirm.^47^ In contrast to ordinary times, there is no prior situation of equality due to the (*pervasive) structural inequality* characterising transitional settings. Additionally, the state is often involved in wrongdoing and thus lacks legitimacy, particularly regarding its authority to impose criminal law and punishment.^48^ Another central critique of a retributivist approach to international crimes is the impossibility of punishing the crime proportionately. Matching punishment to the harm done by collectivities of perpetrators to hundreds or maybe thousands of victims of the gravest crimes would require, as Drumbl notes, abandoning core principles of IHRL in terms of the modality of sanctions or the length of the sentences.^49^ Furthermore, retributivism presupposes that punishment is always necessary, closing the door to other criminal policy considerations and failing in this way to limit the state’s punitive power.^50^

Deterrent rationales have also been criticised in contexts of mass violence. First, there is ‘systematic evidence that severity of punishment (long prison sentences) has negligible marginal deterrent effects’.^51^ This is no different in contexts of mass violence where, additionally, punishments are not applied either because it is practically impossible (due to continuation of the conflict or the selection of cases) or because the authorities are involved in wrongdoing and thus do not apply the law.^52^Moreover, as members of a group targeting other groups due, for example, to their ethnicity, religion, political ideology or race, perpetrators tend to believe that the crimes are justified to achieve a certain goal or simply act according to the group’s norms.^53^ Such individuals can hardly be deterred by any threat of future potential prosecution and punishment.^54^

Furthermore, both deterrence and retributivist theories tend to be offender-centred, being only a response towards the perpetrator or future offenders. This is a significant challenge in transitional contexts since recognising victims, building trust among citizens, restoring and contributing to reconciliation are ordinarily essential goals of TJ. Accordingly, communicative and positive preventive theories have gained attention from scholars focusing on sanctioning international crimes.^55^

### B. Potential of Communication and Positive Prevention

In contrast to retribution or deterrence, under communicative and positive preventive theories, punishment seeks to strengthen the society’s confidence in the rule of law through censuring wrongdoing, as opposed to punishing solely based on just deserts or with the sole aim of deterring future crimes.^56^ In this sense, expressive theories have both backward- and forward-looking purposes.^57^ Furthermore, these theories aim to express social condemnation and confirm the validity of the (criminal) norm through *harsh punishment*^58^ while engaging in a communicative process where censure is expressed in different phases of the criminal process, including the trial and conviction.^59^ If found guilty, the *conviction itself* communicates *censure* to the offender and others by declaring the offender’s responsibility. Thus, punishment is considered the final stage, where ‘most of the messages usually attributed to and communicated by punishment have already been sent by the trial before’.^60^ Therefore, what matters for accountability is not only the punishment itself, but the expressive function of the process more generally.^61^ Hence, for some authors, the fact that communication occurs in different phases of the process gives some flexibility to prosecuting and punishing atrocity crimes in transitional contexts.^62^ Accordingly, we argue that with its focus on communication and censure via the whole criminal process, including trials and verdicts, under expressivism, criminal sanctions can have different modalities extending beyond imprisonment and can therefore include AS.^63^

Expressive theories, furthermore, tend to be less offender-centred than retribution and deterrence. According to Klaus Günther, these theories rediscovered offenders and victims as rational persons and recognised them as communicative agents in the penal system.^64^ Treating victims and offenders as citizens and individuals with agency becomes even more relevant in transitional contexts, where society deals with collective violence, which usually defines certain groups or individuals as enemies or inferior, and thus as appropriate targets for exclusion or abuse.^65^

In addition, the needs and interests of offenders and victims are also considered (to some extent) in the reaction to crime. For instance, criminal law doctrine supports the thesis that resocialisation of the offender is one of the aims of criminal punishment (positive specific prevention).^66^ Moreover, some countries have incorporated reparations to the victim within criminal proceedings as a consequence of the crime.^67^ Some authors even argue that criminal reparations can fulfil the aims of punishment.^68^ Claus Roxin, for example, considers reparative and reconciliatory measures as a new form of sanction which is *independent* of other penalties and corrective measures. The author calls this the ‘Third Route’ (*tercera vía or dritte Spur*) of criminal sanctions. Its autonomy lies upon the fact that the offender must, by themself, collaborate actively to re-establish social peace through repairing the harm done through positive acts and in agreement with the victim.^69^ Criminal reparations, therefore, compensate the offender’s culpability and thus have a direct impact on the custodial penalty, diminishing it, replacing it or extinguishing it. According to Roxin, under the subsidiarity principle, criminal penalties should take a step back when reparations are better suited and sufficient to amend and respond to wrongdoing and contribute to the preventive aims of criminal law.^70^ Pablo Galain argues that voluntary reparation to the victim can be a ‘functional equivalent to penalties’ (*equivalente funcional de la pena*) as long as there is verification and declaration by a court of the offender’s culpability.^71^

In sum, communicating rather than alienating, recognising offenders and victims rather than being offender-centred and including backward- and forward-looking aims are all objectives in line with general TJ goals. Hence, expressive rationales seem to be more apposite than the purely retributive and consequentialist objectives of punishment of atrocity crimes that are sanctioned in such contexts. Arguably, under certain specific conditions related to the design and processes of determining punishment, which we discuss further below, expressivism also offers sufficient flexibility to design appropriate sanctions (beyond imprisonment) that better reflect the needs and goals of transitioning societies.

That being said, there are also concerns about expressive accounts of punishment in such scenarios. First, the message courts generate often can be unclear and fragmented.^72^ Additionally, the message delivers a limited truth centred around individual criminal responsibility instead of collective liability. Secondly, it is questionable that punishment can be justified by its social function in contexts lacking shared normative commitment to the rule of law and basic norms.^73^ The norms’ validity and the legitimacy of the authorities and institutions trying to impose them can be challenged for being part of the abusive apparatus.^74^ Therefore, justifying punishment by its social function cannot be based on affirming pre-existing norms facilitating (directly or indirectly) violence. It would require a *previous or simultaneous* (re)construction of the normative community. This must be done holistically through various judicial and non-judicial mechanisms re-establishing universal core values, such as human dignity and human rights, and transforming pre-existing national legal, social, cultural and economic norms and laws that allowed structural injustice and violence.^75^

Furthermore, for the communicative function of norms and (legal) institutions to work, they must be seen as legitimate, as must the authorities and the processes through which the (re)construction occurs.^76^ Thus, according to Ramji-Nogales, participation in the ‘processes of clarifying and establishing new social norms’^77^ is essential for those communities whose norms are being redefined to trust the authorities and institutions and see them as legitimate. The ‘Norms perceived to be legitimate are significantly more likely to be internalised by the relevant players’.^78^ Arguably, punishment based on expressive rationales has greater potential to be seen as legitimate—as opposed to retributivist and deterrent goals—given its societal focus, ie reinforcing the society’s confidence in the rule of law and preventing future crimes by including offenders (positive specific prevention), victims and broader society (positive general prevention) in the communicative process of judging and punishing crimes. However, to do all that in transitional societies, which by definition require the (re)construction of the normative community, it is necessary for criminal justice (and punishment) to adopt RJ values to promote actual participation of the affected parties in the process of clarifying and establishing new social norms (and not only being message receivers).

As argued in the next section, unlike imprisonment, the nature of AS facilitates such participation. Furthermore, in such challenging contexts, AS may contribute to fostering the interests of transitioning societies while punishing the crimes and thus be justified for international crimes as autonomous sanctions.^79^ To do that, however, AS must also be grounded in expressive rationales, and must adopt stronger RJ values and processes and TJ goals. Without incorporating such values, AS will remain largely inadequate, given that they will take on features and/or be assessed according to the conventional values of the domestic justice system(s) they are part of. Therefore, in the next section, we propose a concept of (re)constructive sanctions, which embrace AS pursuing expressivist goals and integrating strong restorative principles as an adequate punishment for international crimes in transitional settings.

## 4. Moving Towards a New Conceptualisation of Sanctions in Transitional Contexts—Alternative Penalties as (Re)constructive Sanctions

Due to the gravity of international crimes and the fact that they tend to be assessed with the same proportionality metric as ordinary crimes, AS, both at the national and the international level, are usually excluded as autonomous penalties and are imposed either in addition to imprisonment or as a way to suspend, replace or extinguish prison sentences. International criminal justice, for example, has imprisonment as the only self-standing penalty for war crimes, crimes against humanity and genocide. Under the Rome Statute, fines and forfeiture are only possible in addition to imprisonment.^80^ In contrast, this section argues that, under certain conditions, AS as *(re)constructive sanctions* pursuing expressivist goals and adopting RJ principles can be an appropriate punishment in their own right for international crimes in transitional contexts.

RJ focuses on repairing the harm done to victims and restoring the relationship between victims and offenders to allow the reintegration of all parties back into the community.^81^ This is done through an inclusive and dialogical process that, conversely to traditional criminal processes, brings together the affected parties to participate in the resolution of the conflict and promotes the offender to take ‘active responsibility’ in accepting liability and amending the harms caused.^82^ RJ practices may involve punishment, although it does not have a central role.^83^ At the centre is the dialogical process where victims, offenders and the community play an important role in responding meaningfully to wrongdoing and creating responses that will avoid future harm.^84^

In this sense, RJ promotes more ‘constructive punishments’ as alternatives to the infliction of suffering through imprisonment.^85^ Sanctions are more constructive in that they compel the offender to do something positive for the victim and do community work while promoting ‘active responsibility’.^86^ Although active responsibility is found to some extent in other justice models, including traditional criminal justice (eg plea bargain), according to Bueno, Parmentier and Weitekamp, it takes a particular form in RJ: it aims to repair the harm inflicted on victims, offenders and the community and, at the same time, seeks the reintegration of offenders.^87^ Further, ‘a good restorative practice often deals with denial by encouraging those with more minor levels of responsibility to trigger an active responsibility domino effect’.^88^

This approach to accountability and punishment could be advantageous in transitional contexts, given the broader concept of justice promoted by TJ, which is relational and society-focused.^89^ Where there are a large number of perpetrators, victims and affected communities who must live together after mass violence, the goals of regaining trust, transforming relationships and reintegration of offenders become critical.^90^ This is especially so because conflict changes the internal dynamics of communities and often pushes civilians to collaborate in different ways with armed actors or authoritarian regimes. Thus, the lack of trust increases and the family and community bonds are seriously damaged, affecting life in the community.^91^ Therefore, according to Lambourne, ‘a focus on accountability and prosecutions for war crimes and other past human rights abuses that do not rebuild relationships through some kind of restorative process is unlikely to overcome the societal divisions that undermine peace and security’.^92^

Traditional punishment thus becomes undesirable where there are large-scale and systematic human rights violations because it discourages active accountability, excludes offenders from society by imprisoning them^93^ and thus impedes any meaningful engagement between perpetrators and victims.^94^ Additionally, more in a pragmatic sense, a threat of imprisonment can even impede the cessation of violence.^95^ In contrast, if properly designed, AS may allow the end of hostilities and the demobilisation of armed groups, and can work as incentives for perpetrators to acknowledge their responsibility and contribute to the satisfaction of victims’ rights.^96^ Empirical research in Rwanda, for example, has shown that gacaca’s use of community service as punishment, in some instances, encouraged convicted offenders to engage meaningfully with their victims.^97^ Additionally, community service contributed the most to restoration when offenders worked side by side with victims and when the work provided survivors with direct material benefit.^98^Hence, AS can also help repair in some way the harm done and promote spaces for dialogue between victims, offenders and the community, which imprisonment cannot achieve.

Finally, as mentioned above, AS, especially those directed to repair the harm, can be justified by expressivist goals since they may allow the resolution of both the judicial conflict (re-establishment of the normative order) and social conflict (interpersonal relationships) by promoting the resocialisation of the offender (positive specific prevention) and the reparation of the direct victim (positive general prevention).^99^ Although Roxin considers the reparation of the harm as an essential element of the sanctioning system, under his theory, reparations can only substitute or complement traditional penalties. However, if we take the principle of subsidiarity of the penalties seriously, AS should not be seen as mere alternatives to imprisonment but as stand-alone sanctions that can be the first response available for international crimes. This is especially so given the more acute limitations of criminal law and punishment in transitional contexts.

Consequently, there are both practical and principled considerations as to why there is a need to adopt a broader understanding of sanctions deemed appropriate for international crimes, which would allow the imposition of less punitive measures (compared to retributively proportionate imprisonment) to certain categories of perpetrators—or even to those most responsible—in transitional contexts. However, making this case requires not only adopting expressivist goals as justification of punishment, but also much stronger inclusion of RJ principles. Without such inclusion, AS are ill-equipped to address specific TJ goals and thus cannot be deemed adequate to respond to such crimes meaningfully.^100^ Hence, inspired by Roxin’s idea of a *third route*, we propose a third route of sanctions for international crimes in transitional contexts. This third route opens the possibility to conceptualise what we call (re)constructive sanctions.^101^ Given their restorative character, (re)constructive sanctions differ from traditional criminal punishment in their rationales, processes and outcomes.^102^ Hence, they require the reimagining of some conventional criminal law principles. Previously, we have coined the so-called *contextualized principle of proportionality* as a possible tool to assess the adequacy of punishment of international crimes in transitional contexts.^103^ The following subsections further develop the principle and the (re)constructive sanctions.

### A. Values and Justifications

**Figure 1.**
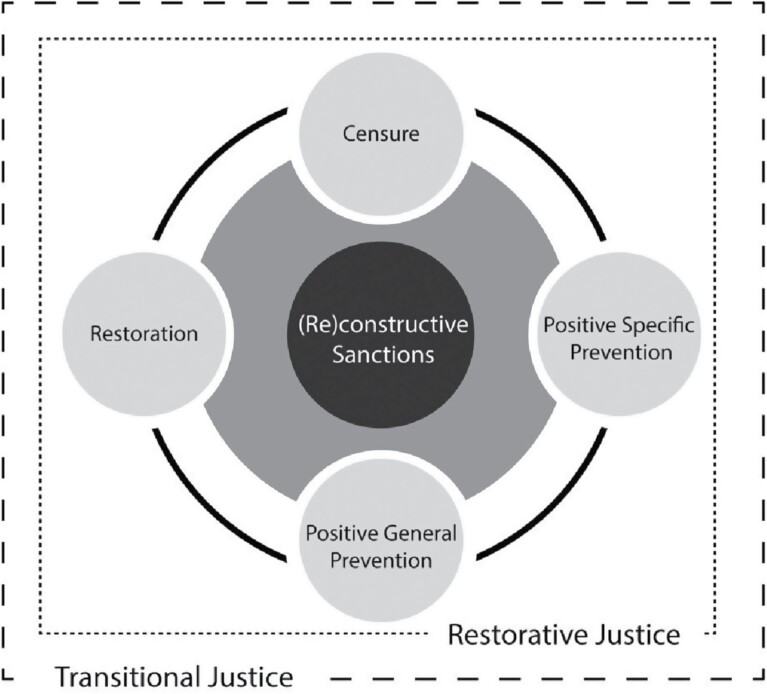
Diagram of the rationales of (re)constructive sanctions, which are shaped by transitional justice goals (long-dashed line) and restorative justice principles and tools (short-dashed line).

As argued above, the *(re)constructive sanctions* proposed here are based on expressive rationales integrating RJ values. Arguably, in combination, these sanctions better reflect the needs and goals of transitional societies. Therefore, the (re)constructive sanctions have four main goals, as shown in Figure 1: (i) expressing censure for the crime committed (through a process declaring the offender’s culpability and imposing burdensome treatment); (ii) positive specific prevention (resocialisation of the offender); (iii) positive general prevention (strengthening the confidence of the rule of law, recognition of victims); and (iv) restoration (reparation of the harm done to victims, (re)construction of relationships).^104^

These sanctions are based on the idea that perpetrators must respond and make amends by recognising the wrongs committed, doing community-based work and other types of reparations which will allow their reintegration into society and the (re)construction of relationships through the meaningful engagement with victims and the community.^105^ Hence, (re)constructive sanctions *combine* burdensome AS, such as community service, (electronic) monitoring and restriction of movement, with symbolic and/or material reparative acts (eg pardon, measures of non-repetition, compensation). The fact that offenders must perform community-based work obliges them to confront directly—and not distantly and isolated, as in prison—the consequences of the wrongs committed. This opens the door for dialogue and thus facilitates offenders’ reintegration into the community, contributing to the fulfilment of restorative and positive specific and general preventive goals. Additionally, making amends to repair the harm done through community-based sanctions potentially provides a more tangible form of justice to victims and affected communities.^106^ The direct benefit to the victims and the community through the offenders’ work and material and symbolic acts of reparation sends a direct message of the recognition of perpetrators’ and victims’ agency and needs, favouring positive general preventive goals. Material and symbolic measures may include events of recognition of the victims and acknowledgement of the wrongs committed, giving information about the remains of disappeared and dead persons, and returning or compensating goods, among others.

Although the appropriate modalities or types of efforts—and their combination—might differ depending on the specific context, the idea is that offenders, by their work and resources, make such amends, as opposed to simply ‘paying’ or ‘compensating’ for the damage caused.^107^ However, such a restorative component of (re)constructive sanctions does not seek the full restitution (*restitutio in integrum*) of the damage caused to victims since they are not reparations *per se* as understood by IHRL.

While encouraging perpetrators to take active responsibility, a restorative communicative approach also compels them to accept liability and demands the fulfilment of several requirements as non-negotiable conditions^108^ for receiving (re)constructive sanctions. Establishing non-negotiable conditions is important to fulfilling TJ’s aims, seeking to ensure accountability and guaranteeing victims’ rights to the truth and reparations. Examples of non-negotiable conditions could be: (i) acknowledgement of responsibility; (ii) contributions to the truth about the crimes committed by the offender and the criminal structure to which the offender belonged;^109^ (iii) making serious efforts to repair the harms caused; and (iv) adopting measures of non-repetition, such as demobilisation and disarmament, or removing the offender from a public position where the crimes were committed.^110^ Complying with such conditions shows perpetrators’ potential for resocialisation and their recognition of the norms they have violated and those being reconstructed as part of the TJ process, serving positive specific and general preventive aims.^111^

Adhering to such non-negotiable conditions may be done in the early stages or during the criminal proceedings. If done in the early stages, the proceedings may be abbreviated. Either way, (re)constructive sanctions must be accompanied by the domestic court’s formal declaration of responsibility of the offender.^112^ Otherwise, the penalties will lose one of their expressive aims, which is to censure the wrongful act.^113^

### B. Processes and Participation in the (Re)constructive Sanctions

The restorative potential of the (re)constructive sanctions also depends on the participation of victims and/or affected communities in their design and proceedings. According to victimological and procedural justice studies, giving victims a voice can be seen as ‘an expression of agency, a means to re-assert one’s standing and receive respect’, and ‘it might also be viewed as an expression of communion, a way to link one’s experience with the criminal proceedings and to connect to other actors’ in and outside the procedure.^114^ Hence, victims should be given the possibility, in either written or oral form, to provide information and evidence related to the crimes while engaging in a more communicative process.^115^ Moreover, victims and communities should be able to express their views concerning the type of work or positive acts that perpetrators will have to complete as part of their sanctions. The restorative nature of these sanctions depends not only on the attitudes of perpetrators, but also on how victims and affected communities perceive them. Only meaningful participation can respond better to victims’ needs, contributing in this way to a greater sense of ownership over the TJ process.^116^ Consequently, having active participation by both victims and offenders in the proceedings is critical, as is the design and implementation of sanctions.

### C. Outcomes: Contextualized Principle of Proportionality as a Means to Assess Proportionality of (Re)constructive Sanctions

Irrespective of their reparative potential, (re)constructive sanctions remain primarily *penal* measures. Nonetheless, as has become clear, some conventional criminal law principles ordinarily applied to punishment, such as proportionality, are largely inadequate to conceptualise and assess such penalties. In societies where the competing ethical, political and practical considerations are eg ending the conflict, contributing to sustainable peace and relational transformation, ‘the question of proportionality and whatever generally agreed-upon criteria might be accepted in normal times appear to be put under severe stress’.^117^ Therefore, transitional contexts and the (re)constructive sanctions require rethinking our existing tools and principles. The *contextualized principle of proportionality* incorporates forward-looking interests of the transition in the assessment of the type and amount of the sanction.

As a general principle of criminal law, proportionality is essential in two aspects: (i) in being, together with the principle of culpability, a limit to excessive punishment; and (ii) in gauging the reprobation for the crime committed.^118^ In this sense, the *contextualized principle of proportionality* retains this limiting function to the punitive power of the state: only those found culpable of a crime can be punished and cannot receive harsher punishment than what they ‘deserve’. However, it requires reimagining how the modality and severity of the sanction are determined and evaluated. Punishment ceases to be based (solely) on ‘just deserts’. Instead, (re)constructive sanctions first and foremost reflect the expressive and restorative aims of punishment and goals of the transitional process (eg satisfaction of victim’s rights, recognition of victims, civic trust, resocialisation, reparation). In other words, the seriousness of the crime is no longer the primary foundation to assess penalties’ proportionality—instead, the assessment centres around restorative and transitional goals.

Consequently, the (re)constructive sanctions will be *directly linked* to the perpetrator’s active responsibility to contribute to the truth, acknowledgement of responsibility and willingness to contribute to societal repair. Accordingly, rather than the seriousness of the crime, these elements will determine how perpetrators will be ‘punished’. If perpetrators do not comply with the imposed conditions, they should not benefit from the (re)constructive sanctions but instead receive more coercive sanctions.^119^

On the other hand, the culpability of the offender and the harm caused by the crime do not lose relevance in assessing the adequacy of (re)constructive sanctions. Their relevance is, however, more *indirect.*^120^ First, doing positive acts to repair the damage done and making efforts to reincorporate into society compensate (although not entirely) the offenders’ culpability.^121^ This means that by amending materially and symbolically the wrong committed, the offender recognises the victim and moves away from the offence, facilitating the offender’s reintegration into society and the reconstruction of relationships.^122^ Furthermore, the relative culpability of an offender, that is, relative to other perpetrators acting in an organisation, can determine the ‘severity’ of the (re)constructive sanction. Thus, the condemnatory aspect of the sanction is reflected in the time and reparative efforts that the perpetrator would need to perform. For instance, more culpable offenders will be committed to these sanctions for a longer period or will do more reparative acts.^123^

Secondly, the relation with the harm is also indirect. More than accounting for the harms in quantitative terms, the sanction is a recognition of the harms through material and symbolic work or activities, seeking to redress the individual and the community. Therefore, adequacy of (re)constructive sanctions is not assessed by whether they ‘quantitatively’ reflect the seriousness of the crime and relating harm, but rather whether they more ‘qualitatively’ reflect the complex and collective character of the wrongdoing and its consequences, including at a community level. In contexts of mass violence, measuring the harms in penal, or even civil, terms is impossible because atrocity crimes are not only predominantly interpersonal offences, but also have collective and political dimensions.^124^ Mass atrocities result in both individual and collective material and immaterial harms, including the rupture of relationships with the victim self and the relationships within families and the community.^125^ The dynamics of mass violence and the rupture of relationships increase mistrust and make life in the community difficult.^126^ Therefore, the harm done extends beyond the harm endured by individual victims and includes ruptured societal structures and relationships.^127^ Accordingly, the character of collective harms should inform the design and modalities of (re)constructive sanctions.

Lastly, (re)constructive sanctions should not oblige the perpetrator in a disproportionate way to their possibilities. While the principle of equality should not be disregarded, the character and modality of the sanctions should also consider the offender’s personal or economic situation.^128^ For instance, the offender may be indigent, and hence may only contribute with symbolic measures and community work, but not with the material resources to perform them or to give as compensation. In contrast, there will be other perpetrators that have the resources to do both.

In sum, the (re)constructive sanctions and the contextualized principle of proportionality proposed here provide an initial framework for assessing the adequacy of AS for mass wrongdoing in terms of rationales, processes and outcomes. First, concerning the *rationales*, the justification of (re)constructive sanctions is grounded in positive general and specific preventive rationales and restorative aims. Therefore, criminal justice processes in transitional contexts should adopt RJ values and practices as complementary tools. In this way, (re)constructive sanctions may help incentivise perpetrators to take active responsibility to respond to and amend the harms done to victims and affected communities. However, to fulfil TJ’s aims, they must be accompanied by non-negotiable conditions seeking to guarantee accountability and the satisfaction of victims’ rights, including a formal declaration of culpability. Secondly, regarding the *processes*, to maximise the sanctions’ restorative goals, they should take part in a communicative restorative process promoting the participation and dialogue between victims and perpetrators during the proceedings and the design and implementation of sanctions. This should always be done respecting the offenders’ due process rights. Finally, regarding the *outcomes*, following the contextualized principle of proportionality, the determination of the sanction and its severity will be linked to the perpetrator’s contribution to achieving TJ goals, to their relative culpability and to how it can qualitatively reflect the complex and collective character of the wrongdoing.

To illustrate the promises, challenges and dilemmas of this approach in the next section, we analyse the case of Colombia—specifically, the sanctioning scheme designed in the FPA. At the time of writing, the SJP has not issued any resolution of conclusions providing for special, reduced or ordinary sanctions. Therefore, we provide a general assessment of the adequacy of sanctions based on the design of the sanctioning scheme rather than on its implementation.

## 5. Colombia’s Special Sanctions as (Re)constructive Sanctions

The TJ system established in the FPA opted for an innovative approach to investigate, prosecute and punish the most serious and representative cases of international crimes through a model seeking to reveal macro-criminal patterns and incentivise offenders to take active responsibility through penal benefits. The penal benefits have already promoted the demobilisation and disarmament of more than 13,000 FARC-EP members.^129^ This demonstrates—on a pragmatic level—the potential that measures other than imprisonment may have in taking the first step towards active responsibility. As mentioned before, the sanctioning regime aims to guarantee the satisfaction of victims’ rights and consolidate peace. The restriction of liberty in delimited geographical areas (for special sanctions) or prison (for reduced or ordinary prison sanctions) has a punitive aim. In particular, the special sanctions have received a lot of attention, given their non-custodial nature and goals. In principle, the rationales, processes and potential outcomes of the special sanctions and the conditionality regime constitute an example of (re)constructive sanctions and shall be assessed accordingly. However, several reservations and challenges concerning their implementation remain.

### A. Justifications and Values

The special sanctions consist of two components: (i) the TOAR (activities, works and actions) with ‘reparative-restorative’ content; and (ii) the effective restriction of rights and liberty in a delimited geographical area.

First, as an example of (re)constructive sanctions, the positive acts are provided in different modalities established in the TOAR, coupled with symbolic reparative measures (eg acknowledgement of responsibility).^130^ The combination of symbolic reparative measures and community-based activities increases their potential to contribute to repairing and rebuilding the relationships and civic trust between offenders, victims and affected communities. In fact, the special sanctions are supposed to be coordinated with other points of the FPA, seeking to establish the conditions for development and equality in the zones most affected by the conflict. Accordingly, for the Colombian Constitutional Court (CCC), the special sanctions must seek to restore the damage done to victims, in particular to end the situation of social exclusion that caused their victimisation, and to reconstruct the social tissue and relationships affected by the conflict.^131^ This clearly shows a broader understanding of the harm by the CCC going beyond the individual dimension and promoting a collective dimension of the harms and sanctions instead.^132^

Secondly, access to the special sanctions depends on fulfilling the conditions contemplated in the conditionality regime.^133^ On the one hand, the conditions of acknowledgement of responsibility, contributing to the ‘complete truth’ about their own and others’ conduct and the circumstances of their commission seek to guarantee the satisfaction of the victim’s rights to truth, recognition, reparations and non-repetition.^134^ Complying with these conditions is key for restorative and positive specific and general preventive goals: the offenders’ participation and recognition of the wrongs committed not only shows their potential for resocialisation, but also their recognition of the victims and the norms that are being constructed, as well as their commitment to amending the harms done. Additionally, they provide valuable information about the facts and people involved in the cases to reveal macro-criminal patterns that might contribute to a better understanding of the conflict, its actors and the root causes that enabled violence.

On the other hand, for the offenders selected to appear before the SJP as part of the macro-cases, attaching special sanctions to certain conditions works as an incentive to take active responsibility and comply with them in a complete and timely manner. Perpetrators can receive special sanctions if they acknowledge their responsibility and contribute with complete truth at the beginning of the proceedings. However, as explained before, if they do not comply with this, or do so only partially or at a later stage in the proceedings, they will receive reduced or ordinary prison sentences. It is important to note that offenders have the option to deny their responsibility and go to the adversarial phase before the SJP’s tribunal. Furthermore, the agreement provides for ‘anticipated TOAR’ (*TOAR anticipados*), which is a tool to promote those offenders who can potentially be called to appear before the SJP to voluntarily repair the damage done to the victims in advance of any proceedings. This means that offenders may do the TOAR *before* the SJP formally imposes them as part of the special sanctions. If offenders are later found responsible, the time and work done through the anticipated TOAR can be considered an early contribution and will thus be reduced from the special sanctions if they satisfy the victims’ rights.^135^ This can work as an incentive to perform early reparative acts that can later be discounted from the number of activities or time imposed as sanctions. Thus, offenders do anticipated TOAR on their own accord, whereas the TOAR, as part of the special sanctions, are imposed by the SJP.

Thirdly, international crimes cannot be amnestied, in contrast to political crimes. This is the first step where communication and censure are adopted by sending a message about the wrongfulness of the crimes committed within the conflict. By judging, deciding on responsibility for the gravest crimes and imposing special sanctions, the SJP sends a message to the victims, society and perpetrators, expressing the wrongfulness of the crimes, the reprobation of such conduct and the recognition of victims as rights bearers, following general preventive aims. As an illustration, in 2021, the SJP issued its first indictments in two macro-cases accusing eight high commanders of the former guerrilla forces, 25 former members of the armed forces and one civilian of war crimes and crimes against humanity.^136^ From those accused, all the ex-FARC-EP high commanders, 21 members of the armed forces and the civilian recognised their responsibility for those crimes. The cases of those denying responsibility were sent to the SJP’s Investigations Unit to be further investigated with a view to opening an adversarial trial.^137^ This is unprecedented in the history of the country.

In sum, the special sanctions can be classified as (re)constructive sanctions since they pursue positive specific and general preventive goals while adopting RJ values. They aim to repair the harm done to the victims and censure the crime committed through a punitive component. They also seek the reintegration of the offenders and (re)construction of relationships, which are essential to consolidate peace. Within the RJ values, they promote the fulfilment of such aims by encouraging offenders to take active responsibility through conditions requiring them to recognise their liability and contribute to the truth.

However, the Colombian case already makes several challenges evident. For instance, delineating the communities where offenders will perform the special sanctions has proven to be particularly complex. The conflict has caused the displacement of nine million individuals within and outside the country over five decades.^138^ Hence, victims are often not living in the communities where they originally lived. Moreover, many offenders committed crimes in different places and regions. These are significant factors that must be at the centre of the design and determination of special sanctions, and must be carefully considered during their implementation.

Concerning the incentives for offenders, there seem to be several additional challenges. First, regarding the anticipated TOAR, it is unclear which individuals, from those subjected to the SJP, will be formally called to appear as accused before the SJP. Thus, offenders might not have sufficient incentives to do them. Secondly, violence continues in several parts of the country, potentially disincentivising offenders and other actors from participating before the SJP and other mechanisms. According to the UN Verification Mission in Colombia, 315 former FARC-EP combatants were murdered up to early 2022.^139^ This is coupled with the lack of support for and fulfilment of what was agreed in 2016 by Duke’s government and its direct attacks against the SJP.

Lastly, it cannot be disregarded that, despite the recognition of responsibility and declaration of culpability of offenders, some constituencies will consider the special sanctions unsuitable, given their non-custodial nature.^140^ Furthermore, although being found responsible, former FARC-EP members may still be allowed to participate in politics. This presents challenges in terms of the messages sent (one of being rewarded) and brings difficulties to the enforcement of sanctions because it is not clear yet how participation in politics will be combined with the implementation of the sanctions.

Despite the practical challenges, there are already some important results in the first phases of the communicative process related to the expressive, restorative and TJ values and aims. The SJP is already sending strong messages of accountability to Colombians and the international community with: (i) the first indictments against members of both the guerrillas and the public forces founding them responsible for international crimes; (ii) the recognition of responsibility by the offenders mentioned; and (iii) the start of adversarial process against those who denied responsibility. Nevertheless, the above-mentioned challenges can impact the sanctions’ goals if not adequately addressed. The security issue is essential not only for the effective implementation of the sanctions, but also, in general, for fulfilling the agreement. Regarding the political participation of former FARC-EP, there needs to be more information on the reasons for their participation.

However, none of these challenges impacts the special sanctions’ classification as (re)constructive sanctions. Notwithstanding, this characterisation also depends on the processes adopted to determine the special sanctions.

### B. Processes and Participation

In terms of the *processes*, the SJP has RJ as a paradigm for its functioning.^141^ For instance, the first phase of the proceedings has a *dialogical* character that promotes dialogue and deliberation between and among victims, their representatives, offenders and the SJP.^142^ Furthermore, it seeks the contribution of information from offenders and victims so the Chambers can balance the information about the facts of the case, the acknowledgement of responsibility and truth, and the imposition of special sanctions.^143^ Victims, individual or collective, may present reports narrating the crimes they have suffered, identifying potential offenders and giving other relevant information about the facts of the crimes. They may also participate in the hearings directly, or through their representatives, by asking questions of and to the defendant or giving testimony.^144^

Another key point is that both victims and perpetrators can also participate in determining suitable sanctions.^145^ Article 141 of Law No 1957 of 2019 establishes that the proposal of sanctions submitted by offenders should be accompanied by mechanisms for consultations with the communities, including indigenous and other ethnic communities, where the sanctions will be executed. In that way, the victims and communities can express their opinion on the suitability of the works or activities that will be carried out. Participation in the sanctions design is *per se* indicative of victims’ agency and is key for ensuring the sanctions’ reparative-restorative goals. The SJP, furthermore, has to adopt adequate measures for groups specially protected by the Constitution, ie people with disabilities, those discriminated against for their sexual orientation, gender, race or ethnicity, or age, among others.^146^ The impact and consequences of mass violence usually differ between these groups. Therefore, adopting this differentiated approach (*enfoque diferencial*) is especially important in designing the special sanctions for amending the harm done and satisfying the victims’ rights, and thus its general preventive aims. Furthermore, sanctions executed in an indigenous community or imposed on its members should be in accordance with the community’s traditions. Interestingly, this might open the door to including some traditional indigenous practices as special sanctions.

Nevertheless, some limitations exist regarding the parties’ participation in the proceedings. One of the challenges of the SJP is to guarantee victims’ effective and equal participation. Given its mandate to investigate macro-cases, there are cases with thousands of registered victims.^147^ Therefore, only some of those victims will be able to participate in a more direct and meaningful way in the hearings and/or creation of reports about the crimes and harms suffered. This may negatively impact other victims’ perceptions about justice being done and thus affect general preventive aims.

Moreover, despite having restorative justice as a paradigm, the SJP is a criminal tribunal where law experts lead the processes rather than the parties. This limits the opportunity for victims and offenders to engage and discuss the offences and the circumstances that gave rise to them, as well as the possible avenues to solving them. Given the criminal nature of the SJP, it must guarantee offenders’ due process rights according to national and international law. This tension between criminal justice principles and restorative approaches is, therefore, one of the main challenges, which are difficult to resolve. However, as Roccatello and Rojas note, ‘restorative justice does not mean sacrificing due process’.^148^ It is up to SJP practitioners to strike a balance between these two competing paradigms.

### C. Outcomes: The Special Sanctions and the Contextualized Principle of Proportionality

The design of the sanctioning scheme indicates that the special sanctions can be deemed adequate according to the contextualized principle of proportionality. First, as previously mentioned, those penal benefits most restrictive of rights and liberties will be imposed on graver acts: political crimes can be amnestied, whereas international crimes cannot. Those found responsible for atrocity crimes will receive special sanctions, reduced or ordinary prison sanctions, where the declaration of responsibility is necessary.^149^

Furthermore, Article 134 of Law No 1957 provides that the content and measure of the sanction will take into consideration: (i) the degree of truth given by the offender and the stage where it was done; (ii) the gravity of the conduct; (iii) the degree of participation and responsibility of the offender, together with aggravating and mitigating circumstances; and (iv) the efforts taken to repair the harm done to victims and to prevent repetition. This provision, therefore, allows determination of how the perpetrator will be sanctioned, depending on their contribution to expressive and restorative aims and TJ goals, rather than based on ‘just deserts’. Additionally, there is a distinction based on the relative culpability of offenders: those who recognise their responsibility and give complete truth concerning the ‘most serious offences’ will receive special sanctions of five to eight years, and those not having a ‘determinative participation’ in those most serious offences will receive between two and five years.

However, the focus on the ‘most serious and representative crimes’ and the distinction of those with ‘determinative participation’ have been seen as problematic by some constituencies, arguing that it does not seem to comply with ICL and IHL standards.^150^ For example, some civil organisations did not agree with the selection strategy, which meant that not all human rights violations would be investigated and prosecuted. The OTP and some scholars have also noted the ambiguity of the modes of liability, particularly of ‘determinative participation’, which may lead to a waiver of criminal prosecution for individuals liable for serious contributions to grave crimes, even if indirectly or by culpable omission.^151^ Additionally, the definition of command responsibility established in the FPA seems to be more limited than the one established by the Rome Statute, which provides a broader concept of *mens rea* and does not require effective control over the specific conduct.^152^ Ideally, the modes of liability provided at the national level should be clear and in accordance with international standards. Otherwise, this could affect positive general prevention, given that those with greater responsibility could avoid receiving special sanctions.

In addition to that, other challenges related to the implementation of special sanctions and thus assessment of their adequacy are that: (i) there are still no clear rules on how the SJP will measure an offender’s contribution to the truth or their efforts to amend the harm to victims and the communities; (ii) it is unclear how harms will be assessed or how levels and characters of harm will be taken into consideration for proportionality purposes when determining the sanctions; and (iii) the SJP cannot issue economic compensations;^153^ thus, victims should be given enough information clarifying that. Communicating effectively what the special sanctions are and what the SJP does is essential to managing expectations.

Consequently, some conceptual points remain unclear and some rules indicating how special sanctions will be implemented in practice are missing. These are essential for assessing the adequacy of the special sanctions under the contextualized principle of proportionality. The SJP is starting to show some results. Yet, without any sanctions being determined or issued so far, it is difficult to make a more thorough assessment regarding the actual determination of sanctions. Notwithstanding, we believe that, overall, the design of the sanctioning regime to determine penalties is in accordance with the contextualized principle of proportionality. Whether the imposition of these sanctions will be adequate will depend on the implementation of the sanctioning regime in particular cases and how the SJP will further operationalise the innovative principles contained in the sanctioning regime.

To conclude, in general, the sanctioning regime under the FPA constitutes an example of (re)constructive sanctions with their specific justifications, processes and assessment of their adequacy. Thus, despite their leniency, the special sanctions seem adequate for punishing international crimes as part of Colombia’s transitional process. Given their unprecedented character and generally retributive orientation of Colombian criminal law, however, the legitimacy of this sanctioning regime has been questioned and, as such, can threaten its successful implementation. There is no doubt that this unique sanctioning scheme with ambitious goals may be an example, at least in theory, for future peace agreements and TJ processes in different contexts. However, it remains to be seen how the sanctioning scheme will operate in practice.

## 6. Conclusions

The point of departure of this article is that criminal law and harsh punishment, such as imprisonment, should not be the primary response to crimes committed in contexts of mass violence, given its numerous limitations in such circumstances. International crimes and grave human rights violations are likely to occur in contexts characterised by pervasive structural inequalities and social injustices, which are the product of broader social, political, economic, cultural and historical factors. Therefore, punishing perpetrators with conventionally conceived, proportional prison sentences does not address the root causes enabling the violence and is thus unlikely to deter or prevent future crimes.

While criminal justice may still play an important role in reinforcing the rule of law, dominant punishment rationales and traditional penalties are largely ill-suited for sanctioning international crimes in transitional contexts. Criminal prosecutions and punishment, based on purely retributivist or deterrence justifications, may prevent putting an end to violence and make macro-criminal structures hold on to power. Moreover, proportional prison penalties discourage active responsibility-taking, banish offenders, disaffect victims by not relating to their needs and impede any meaningful engagement between perpetrators and victims.

Consequently, given the moral, legal and practical challenges of sanctioning mass atrocities in transitional contexts, our understanding of punishment and its basic principles needs rethinking. Such reconceptualisation envisages a more collectively oriented focus in designing and evaluating punishment, which is both (i) past- and future-oriented and (ii) offender- and victim-centred. Inspired by the case of Colombia, we develop the *(re)constructive sanctions* as penal measures to sanction mass atrocities aligned with TJ goals and incorporating RJ elements, which seek to encourage active responsibility taking, repair the harm done to victims and communities, reintegrate offenders into society and reconstruct societal relationships. We argue that (re)constructive sanctions comply with expressive punishment rationales and contribute to achieving TJ goals if properly designed and implemented. Such penalties can thus be considered adequate punishment for international crimes as long as they are designed as part of a communicative restorative process and are accompanied by certain non-negotiable conditions to satisfy victims’ rights.

While in some situations (re)constructive sanctions may be a better response for past wrongdoing, they can also face many challenges. To maximise their expressive and reconstructive capacity, they should be part of a holistic policy addressing the different political and socio-economic problems at the roots of the conflict. As Colleen Murphy has stated, ‘the expressive meaning of responses to wrongdoing is not fixed but is shaped by other responses to wrongdoing’.^154^ Future empirical and theoretical research can explore and discuss the potential of these sanctions in different transitional contexts.

## Footnotes

1 Isabella Bueno and Andrea Diaz, ‘Which Approach to Justice in Colombia under the Era of the ICC’ (2013) 13 International Criminal Law Review 211, 211–15.

2 Colombian Unit for the Attention and Integral Reparations for Victims <https://www.unidadvictimas.gov.co/es/registro-unico-de-victimas-ruv/37394> accessed 26 June 2021.

3 United Nations press release, ‘Secretary-General Congratulates Colombia on Second Anniversary of Peace Agreement Launch, Says Hard Work Ahead to Consolidate Early Gains’ (23 November 2018) <https://press.un.org/en/2018/sgsm19362.doc.htm> accessed 5 February 2021. In addition, in 2021 the Members of the UN Security Council reiterated their full and unanimous support for the peace process in Colombia. Security Council Press Statement on Colombia, SC/14425 (28 January 2021) <https://press.un.org/en/2021/sc14425.doc.htm> accessed 5 February 2021.

4 Courtney Hillebrecht, Alexandra Huneeus and Sandra Borda, ‘The Judicialization of Peace’ (2018) 59 Harv Int’l LJ 279.

5 Final Peace Agreement to End the Conflict and Establish a Stable and Long-lasting Peace, 24 November 2016 (FPA).

6 FPA, 164–5; Ley No 1957 de 2019, arts 125–7 and 141.

7 This article uses the terms ‘atrocity crimes’, ‘international crimes’, ‘mass atrocities’ or ‘mass violence’ interchangeably in referring to collective, large-scale, organised violence that can legally be qualified as genocide, crimes against humanity or war crimes.

8 This article understands alternative or intermediate sanctions as punishment which falls between probation and prison, including community service, house arrest, intensive supervision programmes, electronic monitoring and reparative acts within the criminal justice system. See Norval Morris and Michael Tonry, *Between Prison and Probation: Intermediate Punishments in a Rational Sentencing System* (OUP 1990) 4ff.

9 Beatriz E Mayans-Hermida and Barbora Holá, ‘Balancing “the International” and “the Domestic”: Sanctions under the ICC Principle of Complementarity’ (2020) 18 JICJ 1103, 1106–22.

10 ibid; Natalie Sedacca, ‘The “Turn” to Criminal Justice in Human Rights Law: An Analysis in the Context of the 2016 Colombian Peace Agreement’ (2019) 19 Human Rights Law Review 315, 330–1; Nicolás Carrillo-Santarelli, ‘The Pragmatism of Justice: On the International Lawfulness and Legitimacy of Alternative Sanctions’ (2020) 44 DPCE 3117, 3129 <www.dpceonline.it/index.php/dpceonline/article/view/1145> accessed 13 December 2020.

11 Cooperation Agreement Between the Office of the Prosecutor of the International Criminal Court and the Government of Colombia, 28 October 2021, 3 <https://www.icc-cpi.int/sites/default/files/itemsDocuments/20211028-OTP-COL-Cooperation-Agreement-ENG.pdf> accessed 28 February 2022.

12 This article conceptualises perpetrators as individuals that in one way or another are involved in atrocities.

13 James Stewart, Deputy Prosecutor of the ICC, ‘The Role of the ICC in the Transitional Justice Process in Colombia’ (ICC, 30 May 2018) paras 141–51 <https://www.icc-cpi.int/news/role-icc-transitional-justice-process-colombia-speech-james-stewart-deputy-prosecutor-icc> accessed 28 May 2021.

14 Mayans-Hermida and Holá (n 9) 1125ff.

15 FPA.

16 Maria Paula Saffon, ‘The Colombian Peace Agreement: A Lost Opportunity for Social Transformation?’ in Jorge L Fabra-Zamora and others (eds), *The Colombian Peace Agreement: A Multidisciplinary Assessment* (Routledge 2021) 71.

17 Mark Freeman and Iván Orozco, *Negotiating Transitional Justice: Firsthand Lessons from Colombia and Beyond* (CUP 2020) 73.

18 ibid.

19 ibid.

20 Ley No 1957, arts 125–43.

21 ibid arts 125 and 141.

22 ibid art 141.

23 Jurisdicción Especial para la Paz, ‘Lineamientos en materia de sanción propia y Trabajos, Obras y Actividades con contenido Reparador-Restaurador’, Sección de Reconocimiento de Verdad y Responsabilidad Tribunal para la Paz, 5–8 <https://www.jep.gov.co/Sala-de-Prensa/Paginas/Conozca-Los-lineamientos-en-materia-de-sanción-propia-y-Trabajos,-Obras-y-Actividades-con-contenido-Reparador---Restaurador.aspx> accessed 16 October 2020.

24 Ley No 1957, art 141.

25 ibid arts 126 and 129. For an explanation and discussion on the term ‘determinative participation’, see Sabine Michalowski, Michael Cruz and Hobeth Martínez, *¿A quiénes sancionar? Máximos Responsables y Participación Determinante en la Jurisdicción Especial para la Paz* (DeJuSticia 2020) <https://www.dejusticia.org/publication/a-quienes-sancionar/> accessed 7 February 2021.

26 Ley No 1957, arts 128–30.

27 Lily Rueda Guzmán and Barbora Holá, ‘Punishment in Negotiated Transitions: The Case of the Colombian Peace Agreement with the FARC-EP’ (2019) 19 International Criminal Law Review 127, 147.

28 For a similar opinion, see Hillebrecht (n 4) 321–2.

29 Mark Drumbl, *Atrocity, Punishment and International Law* (CUP 2007) 150ff; Sergey Vasiliev, ‘Punishment Rationales in International Criminal Jurisprudence: Two Readings of a Non-question’ in Florian Jeßberger and Julia Geneuss (eds), *Why Punish Perpetrators of Mass Atrocities? Purposes of Punishment in International Criminal Law* (CUP 2020) 72ff.

30 Gerlach coined the concept of ‘extremely violent societies’, ‘which seeks to deconstruct conventional understandings of genocidal mass violence and to re-contextualize it within a larger framework of conflict and in the grassroots nature of the other types of violence from which these events emerge’. Cited by Susanne Karstedt, ‘Contextualizing Mass Atrocity Crimes: The Dynamics of “Extremely Violent Societies”’ (2012) 9 European Journal of Criminology 499, 499.

31 Roelof Haveman and Alette Smeulers, ‘Criminology in a State of Denial—Towards a Criminology of International Crimes: Supranational Criminology’ in Alette Smeulers and Roelof Haveman (eds), *Supranational Criminology: Towards a Criminology of International Crimes* (Intersentia 2008) 14.

32 Susanne Karstedt, ‘Contextualizing Mass Atrocity Crimes: Moving Toward a Relational Approach’ (2013) 9 Annual Review of Law and Social Science 383, 386; Stefan Harrendorf, ‘How Can Criminology Contribute to an Explanation of International Crimes?’ (2014) 12 JICJ 231ff.

33 Dawn L Rothe and Christopher W Mullins, ‘Genocide, War Crimes and Crimes against Humanity in Central Africa: A Criminological Exploration’ in Smeulers and Haveman, *Supranational Criminology* (n 31) 143; Frank Neubacher, ‘Criminology of International Crimes’ in Jeßberger and Geneuss (n 29) 33.

34 Alette Smeulers and Roelof Haveman, ‘International Crimes and Criminology: An Agenda for Future Research’ in Smeulers and Haveman, *Supranational Criminology* (n 31) 489.

35 Neubacher (n 33) 32, 37–8; Rothe and Mullins (n 33) 150–2.

36 Margaret M deGuzman, ‘Harsh Justice for International Crimes?’ (2014) 39 Yale J Int’l L 1, 2; Jens D Ohlin, ‘Towards a Unique Theory of International Criminal Sentencing’ in Goran Sluiter and Sergey Vasiliev (eds), *International Criminal Procedure: Towards a Coherent Body of Law* (Cameron May 2009) 390.

37 Stephan Parmentier, Kris Vanspauwen and Elmar Weitekamp, ‘Dealing with the Legacy of Mass Violence: Changing Lenses to Restorative Justice’ in Smeulers and Haveman, *Supranational Criminology* (n 31) 338–43.

38 Pablo de Greiff, ‘Theorizing Transitional Justice’ in Kieran McEvoy and Louise Mallinder (eds), *Transitional Justice: Critical Concepts in Law*, vol 1 (Routledge 2017) 58.

39 Wendy Lambourne, ‘Transitional Justice and Peacebuilding after Mass Violence’ (2009) 3 International Journal of Transitional Justice 28, 30.

40 Jaya Ramji-Nogales, ‘Designing Bespoke Transitional Justice: Pluralist Process Approach’ (2010) 32 Mich J Int’l L 1, 69ff.

41 Colleen Murphy, *The Conceptual Foundations of Transitional Justice* (CUP 2017) 21–37; Paul Gready and Simon Robins, ‘From Transitional to Transformative Justice’ in McEvoy and Louise Mallinder (n 38); Dustin Sharp, ‘What Would Satisfy Us? Taking Stock of Critical Approaches to Transitional Justice’ (2019) 13 International Journal of Transitional Justice 570, 578ff.

42 De Greiff, ‘Theorizing Transitional Justice’ (n 38).

43 Gready and Robins (n 41) 93ff.

44 Murphy (n 41) 84–95. According to Murphy, transitional societies are characterised by certain circumstances that make them distinct from stable democracies and thus require different responses to past violence and abuses. Among these circumstances, Murphy identifies the *normalised collective* and *political* wrongdoing; the existing *pervasive structural inequalities* that fail to recognise the equal status of some individuals or groups; the *serious existential uncertainty* on whether the transition to democracy will occur; and the *fundamental uncertainty about authorities*, which are generally complicit in wrongdoing.

45 ibid.

46 Wendy Lambourne, ‘Restorative Justice and Reconciliation: The Missing Link in Transitional Justice’ in Kerry Clamp (ed), *Restorative Justice in Transitional Settings* (Routledge 2016) 59.

47 Murphy (n 41) 84–96.

48 ibid.

49 Mark Drumbl, ‘We’re Exhausting Ourselves, Let’s Get Busy Instead: A Comment on the Contributions by Jakob VH Holtermann, Mota Kremnitzer and Daniela Demko’ in Jeßberger and Geneuss (n 29) 198.

50 Claus Roxin, *Problemas básicos del Derecho Penal* (Diego Manuel Luzon Peña, Reus 1976) 14–15. There are various versions of retributivism: (i) positive, which holds that those guilty *must always be punished* to the full extent of their desert; and (ii) negative, which uses just desert as a constraint against too severe punishment, allowing downward deviations. For an in-depth study, see Jesper Ryberg, *The Ethics of Proportionate Punishment: A Critical Investigation* (Kluwer Academic Publishers 2004).

51 Karstedt, ‘Moving Toward a Relational Approach’ (n 32) 395. Criminologists argued that it is the probability of being caught, not the harsh punishment, which deters perpetrators.

52 Neubacher (n 33) 29.

53 Alex Alvarez, ‘Destructive Beliefs: Genocide and the Role of Ideology’ in Smeulers and Haveman, *Supranational Criminology* (n 31) 213.

54 Smeulers and Haveman, ‘International Crimes and Criminology’ (n 34) 489–90; Phil Clark, *The Gacaca Courts, Post-Genocide Justice and Reconciliation in Rwanda: Justice without Lawyers* (CUP 2011) 351. For a different view on deterrence, see Jakob v Holtermann, ‘Can I Be Brought before the ICC?: Deterrence of Mass Atrocities between Jus in Bello and Jus ad Bellum’ in Jeßberger and Geneuss (n 29).

55 Carsten Stahn, *Justice as a Message: Expressivist Foundations of International Criminal Justice* (OUP 2020) 40; Robert Sloane, ‘The Expressive Capacity of International Punishment: The Limits of the National Law Analogy and the Potential of International Criminal Law’ (2007) 43 Stan J Int’l L 39.

56 Drumbl, *Atrocity, Punishment* (n 29) 173. According to Kai Ambos, communicative theories (from a common-law tradition) and positive general prevention theories (from continental criminal law doctrine) are based on the same concept, namely, strengthening the confidence in the rule of law through punishment. See Kai Ambos, *Treatise of International Criminal Law, Volume I: Foundations and General Part* (2nd edn, OUP 2013) fn 162.

57 There are several theories falling under the expressive and general prevention umbrellas. Some of them are considered as mixed theories, but others are not. For example, some consider Duff’s communicative theory not a mixed theory because, as opposed to retributivist theories, his approach assigns a further aim to punishment: ‘to enter into a moral dialogue … with the offender’, since ‘the possibility of moral repentance and reform depends on how the offender understands and responds to the punishment’. See Anthony Duff and David Garland, ‘Introduction: Thinking about Punishment’ in Anthony Duff and David Garland (eds), *A Reader on Punishment* (OUP 1994) 15.

58 Joel Feinberg, however, has noted that ‘it is social disapproval and its appropriate expression that should fit the crimes, and not hard treatment (pain) as such’. Joel Feinberg, ‘The Expressive Function of Punishment’ (1965) 49 The Monist 397, 423.

59 Anthony Duff, *Punishment, Communication and Community* (OUP 2001) ch 3. Expressive theories differ on the purpose of the message that punishment attempts to transmit. For example, it can express social condemnation (Feinberg) or censure to the wrongdoer (Von Hirsh), or it can teach the offender a moral lesson or induce her to repentance, reform and reconciliation with the community (Duff). See Duff and Garland, *A Reader on Punishment* (n 57) 14ff.

60 Klaus Günther, ‘Positive Prevention and the Idea of Civic Courage in International Criminal Law’ in Jeßberger and Geneuss (n 29) 214.

61 For most versions of communicative and expressivist theories (from a common law tradition), punishment is a necessary but insufficient element. However, for some positive general prevention theories, punishment might be deserved but not necessary. For a discussion on punishment theories, see Tatjana Hörnle, *Teorías de la pena*, Universidad Externado de Colombia, Centro de Investigación en Filosofía y Derecho, Colección de Estudios No 43 (Digiprint Editores 2015).

62 Elena Maculan and Alicia Gil, ‘The Rationale and Purposes of Criminal Law and Punishment in Transitional Contexts’ (2020) OJLS 1, 16–20.

63 Given the lack of evidence showing the improvement of public safety by the use of imprisonment and it being counterproductive in the reintegration of offenders, AS have been introduced in various jurisdictions around the world as appropriate penal measures for ordinary crimes (United Nations Office on Drugs and Crime, *Handbook of Basic Principles and Promising Practices on Alternative to Imprisonment* (2007) 3ff). Like imprisonment, AS have a punitive element since they involve the limitation of freedom and rights (Joan Petersilia and Elizabeth Piper Deschenes, ‘Perceptions of Punishment: Inmates and Staff Rank the Severity of Prison versus Intermediate Sanctions’ (1994) 74 The Prison Journal 308). Nevertheless, they have broader objectives, such as offender reintegration and promoting accountability by allowing offenders to remain in the community (Brian Johnson and Stephanie Dipietro, ‘The Power of Diversion: Intermediate Sanctions and Sentencing Disparity under Presumptive Guidelines’ (2012) 50 Criminology 811, 842). In transitional contexts, with their focus on reconstructing social cohesion after mass violence, these goals become even more salient, as argued in section 4.

64 Klaus Günther, ‘Criminal Law, Crime and Punishment as Communication’ (2014) Normative Orders Working Paper, Cluster of Excellence at Goethe University Frankfurt/Main 2014-02, 4–5 <http://publikationen.ub.uni-frankfurt.de/frontdoor/index/index/docId/34663> accessed 3 September 2020. This does not mean, however, that victims have a right to punishment. In this we agree with Maculan, Gil and Galain. According to them, victims’ satisfaction should not be considered a superior purpose to other preventive aims, nor can they replace the main goal of criminal law, which is to protect the social order and legal interests of the community. See Maculan and Gil (n 62) 5–9; Pablo Galain, *La reparación del daño a la víctima del delito* (Tirant lo Blanch 2010) 88–90.

65 Murphy (n 41).

66 Yesid Reyes-Alvarado, ‘Victimas, fin y necesidad de la penal en el Derecho penal y en la llamada justicia transicional’ in Alicia Gil and Elena Maculan (eds), *La Influencia de las Víctimas en el Tratamiento Jurídico de la Violencia Colectiva* (Dykinson 2017) 196ff.

67 Galain (n 64) 168. There is no consensus in the criminal law doctrine about the inclusion of reparations as an autonomous form of sanction within criminal law.

68 ibid 99–113; Geoffrey Sayre-McCord, ‘Criminal Justice and Legal Reparations as an Alternative to Punishment’ (2001) 11 Philosophical Issues: Social, Political and Legal Philosophy 502; Lucia Zender, ‘Reparation and Retribution: Are They Reconcilable?’ (1994) 57 MLR 228.

69 Claus Roxin, ‘Pena y reparación’ (1999) 52 Anuario de Derecho Penal y Ciencias Penales 5. Security measures and penalties are considered as the First and Second Routes.

70 ibid 13. Roxin justifies the inclusion of reparations as a third route on the principles of subsidiarity and *ultima ratio*. From a criminal policy point of view, the notion of responsibility relates to the necessity to punish in the concrete case. Thus, imposing a penalty might not be necessary if the offender has shown, through positive reparative acts, that the preventive punishment aims are fulfilled. In Roxin’s theory, however, the declaration of responsibility is not necessary.

71 Galain (n 64) 342.

72 Ramji-Nogales (n 40) 8–9; Drumbl, ‘We’re Exhausting Ourselves’ (n 49) 202–5.

73 Rosa Ehrenreich Brooks, ‘The New Imperialism: Violence, Norms, and the “Rule of Law”’ (2003) 101 Mich L Rev 2275, 2322–3. Brooks notes that ‘In some societies that lack a shared normative commitment to the rule of law, only nonlegal initiatives are likely to have any immediate effect on the degree and distribution of violence—and they will be effective only if they succeed in offering alternative narratives that assign new cultural meaning to violence and suffering.’

74 David Gray, ‘Extraordinary Justice’ (2010) 62 Ala L Rev 55, 101.

75 Sharp (n 41).

76 Ramji-Nogales (n 40) 11–21.

77 ibid 8.

78 ibid 11–12.

79 cf Mark Drumbl, ‘Alternatives to Custodial Sentences’ in Róisín Mulgrew and Denis Ables (eds), *Research Handbook on the International Penal System* (Edward Elgar Publishing 2016) 384.

80 ICC Statute, art 77(2).

81 Róisín Mulgrew, *Towards the Development of the International Penal System* (CUP 2013) 224.

82 ibid.

83 Michael Wenzel and others, ‘Retributive and Restorative Justice’ (2008) 32 Law & Hum Behav 375, 377; John Braithwaite, ‘Between Proportionality & Impunity: Confrontation, Truth, Prevention’ (2005) 43 Criminology 283, 297.

84 Kerry Clamp, ‘Clearing the Conceptual Haze: Restorative Justice Concepts in Transitional Settings’ in Clamp, *Restorative Justice* (n 46) 20.

85 Wenzel and others (n 83) 377.

86 ibid.

87 Isabella Bueno, Stephan Parmentier and Elmar Weitekamp, ‘Exploring Restorative Justice in Situations of Political Violence’ in Clamp, *Restorative Justice* (n 46) 38.

88 Braithwaite (n 83) 291. Moreover, dealing with denial through active responsibility may be even more critical in TJ because it can allow a broader understanding of the collective dimension of such crimes and the different actors involved.

89 Although RJ emerged as a response to the limitations and failures of criminal justice systems operating in ordinary times, rather than as a new paradigm to address mass wrongdoing, it shares some goals sought by TJ, eg reparation of victims and affected communities, reintegration of the offender, reconciliation and reconstruction of relationships. Therefore, RJ, as a complementary paradigm, may contribute with the values it seeks to promote and the processes and outcomes through which it promotes them. For a discussion, see Clamp, ‘Clearing the Conceptual Haze’ (n 84).

90 Lambourne, ‘Restorative Justice’ (n 46) 59.

91 Julieta Lemaitre Ripoll, *El Estado siempre llega tarde: La reconstrucción de la vida cotidiana después de la guerra* (Siglo Veintiuno Editores 2019) 19, 45–56.

92 Lambourne, ‘Transitional Justice’ (n 39) 38–9.

93 Braithwaite (n 83). According to Braithwaite, ordinary criminal justice usually promotes passive responsibility, which is backward-looking in that it seeks to be just in the way that it makes the wrongdoer suffer, resulting in a culture of denial due to the fear of being punished.

94 Clark (n 54) 353.

95 Bueno, Parmentier and Weitekamp (n 87) 50.

96 The Colombian case is a good example of this. Both its Peace and Justice Law and the FPA allowed the demobilisation and disarmament of paramilitary groups and FARC members, respectively, through reduced sentences and AS.

97 Clark (n 54) 353. In Rwanda, for instance, community service was used for some offenders who admitted their crimes before the gacaca system. However, some victims viewed these sanctions as too lenient and some did not want to engage with the perpetrators due to fear.

98 ibid.

99 Roxin, ‘Pena y reparación’ (n 69).

100 The Justice and Peace Law is an example of this. Despite allowing AS, consisting in reduced prison sentences extinguishing the primary prison sentence, it was heavily criticised at the beginning for giving benefits to paramilitary members without sufficiently guaranteeing victims’ rights.

101 We call them (re)constructive sanctions because one of its main goals is to reconstruct in new ways or construct anew the relationships between offenders, victims and the community, based on the new norms being established. It is not about restoring, since in contexts of mass violence relationships are unequal. Nor is it about reaffirming previous laws that promoted structural inequalities.

102 The third route proposed here is inspired by Roxin’s theory but is not the same. First, the (re)constructive sanctions are not traditional AS nor criminal reparations. Rather, they include a restorative rationale apart from the expressivist ones, meaning that RJ is one element to be considered when designing and imposing AS. Secondly, in contrast to Roxin’s theory, (re)constructive sanctions can be imposed for the gravest crimes as autonomous penalties and not simply to mitigate prison penalties. Like Roxin’s proposal, it uses the category of culpability to assess whether in the concrete case it is necessary to punish with traditional penalties or (re)constructive sanctions. However, this culpability principle takes into consideration forward-looking aims of the transition. The third route proposed in this article also defers from the ‘tercera vía’ proposed by Cote and Avella-Herrera. The authors note that transitional societies generally impose blanket amnesties and pardons or retributive measures. Between these two ‘extremes’ they propose the category of ‘transitional restorative justice’ as a ‘tercera vía’, which ‘is autonomous between transitional and restorative justice’ and allows to broaden the tools that judicial authorities can use in complex cases. See: Silvia-Juliana Cote and Mónica-Andrea Avella-Herrera, ‘La autonomía de la justicia transicional restaurativa’ in Luis-Miguel Gutiérrez-Ramírez (ed) *Las Sanciones en la Justicia Transicional Restaurativa y los Desafíos de la Jurisdicción Especial para la Paz en Colombia* (Ibañez, 2021).

103 It is beyond the scope of this article to do a dogmatic development. This is only a first step to reimagining such principles and goals in transitional contexts.

104 These four principles should feature in three stages: (i) the design of institutions addressing international crimes; (ii) when judges decide the mode and severity of the sanction imposed in the particular case; and (iii) in the enforcement of sanctions. Concerning the first stage, the principles should be taken into consideration by the legislature when determining *in abstracto* the sanctions that will be available to punish international crimes.

105 In transitional contexts, delineating the community is often not straightforward. As the Colombian case shows below, there are many challenges related to this. The challenges increase when there are foreign inputs into the crimes committed.

106 Conor McCarthy, ‘The International Criminal Court’s Regime of Victim Redress: Non-punitive Responses to Crimes under the Rome Statute’ in Mulgrew and Ables (n 79) 414.

107 Sayre-McCord (n 68) 505. Although there is flexibility in determining the modalities, types of efforts or conditions that perpetrators must fulfil, they should comply with basic human rights and international standards on due process, impartiality, independence, etc. Further, the sanctions should be regulated and accompanied by oversight mechanisms.

108 Mulgrew (n 81) 225–6.

109 The trial and (re)constructive sanctions should not be seen as truth-finding mechanisms. The concern of criminal trials is the judicial truth, which has a limited focus. Although (re)constructive sanctions promote active accountability, acknowledging responsibility and contributing to the truth as part of the criminal process, they are limited in this sense. Furthermore, it is important to keep in mind that giving or disputing an account of truth in transitional contexts often comes with political antagonism. On this, see Marti Koskenniemi, ‘Between Show Trials and Impunity’(2002) 6 Max Planck Yearbook of United Nations Law 1.

110 Rodrigo Uprimny and Maria Paula Saffón, *Transitional Justice, Restorative Justice and Reconciliation. Some Insights from the Colombian Case* (DeJuSticia 2017) <https://www.dejusticia.org/wp-content/uploads/2017/04/fi_name_recurso_55.pdf> accessed 13 December 2020.

111 The example of the FARC-EP is illustrative on this point. There were previous attempts to reach peace with this guerrilla group; however, they did not recognise the Colombian Constitution. With the FPA, they recognised it together with the institutions created by it.

112 Ideally, investigations and trials should be carried out against those most responsible, mid- or high-level perpetrators, and low-level perpetrators liable for serious contributions to international crimes pertaining to all the groups involved in atrocity crimes.

113 In this we agree with Galain, who argues that there needs to be a formal declaration of culpability.

114 Anthony Pemberton, Puline GM Aarten and Eva Mulder, ‘Beyond Retribution, Restoration and Procedural Justice: The Big Two of Communion and Agency in Victims’ Perspective on Justice’ (2017) 23 Psychology, Crime & Law 682, 684. According to the authors, ‘communion concerns social relatedness, warmth, expressiveness and affiliation, while agency concerns individual striving, competence, power and instrumentality’. Furthermore, they explain that both offenders and victims experience impairment to their sense of communion with others.

115 This could be done through the recognition of victims as a party to the procedure or through the possibility of expressing their views, even if they are not recognised as a party as such.

116 Mijke de Waardt and Sanne Weber, ‘Beyond Victims Mere Presence: An Empirical Analysis of Victim Participation in Transitional Justice in Colombia’ (2019) 11 Journal of Human Rights Practice 210, 211ff. The authors, however, warn about participation of victims that is not meaningful.

117 Paul Seils, *Squaring Colombia’s Circle: The Objectives of Punishment and the Pursuit of Peace* (International Centre for Transitional Justice, June 2015) 13 <www.ictj.org/publication/squaring-colombia-circle-objectives-punishment-peace> accessed 25 October 2020.

118 Santiago Mir Puig, *Derecho Penal Parte General* (10th edn, Editorial Reppertor 2016) 42–3.

119 This is similar to Braithwaite’s RJ pyramid, which includes restorative, deterrent and incapacitative justice. The pyramid has RJ options as a starting point and incapacitation as the endpoint. The idea is that coercive control may be seen as more legitimate only when more dialogic and respectful forms of engagement and punishment have been tried first. See John Braithwaite, *Restorative Justice and Responsive Regulation* (OUP 2022) ch 2.

120 Galain (n 64) 150–1 explains proportionality for reparative acts in ordinary criminal justice systems.

121 Roxin, ‘Pena y reparación’ (n 69) 9–10. The principle of culpability here is related to the necessity of the penalty/punishment in the concrete case, rather than solely with the censure of the crime. This allow the inclusion of forward-looking aims, which in this case are preventive and restorative. cf Reyes-Alvarado (n 66) 197–201.

122 ibid.

123 This is similar to the principle of gradation that has been used at the International Criminal Tribunal for the former Yugoslavia and International Criminal Tribunal for Rwanda. For an explanation of said principle, see Barbora Holá, *International Sentencing ‘A Game of Russian Roulette or Consistent Practice?’* (Uitgeverij BOXPress 2012) 40–2.

124 Pablo de Greiff, ‘Justice and Reparations’ in Pablo de Greiff (ed), *The Handbook of Reparations* (OUP 2006).

125 Anthony Pemberton and Rianne Letschert, ‘Victimology of Atrocity Crimes’ in Barbora Holá and others (eds), *The Oxford Handbook of Atrocity Crimes* (OUP 2022) 11.

126 Lemaitre Ripoll (n 91) ch 1.

127 Stahn (n 55) 366.

128 Galain (n 64) 150–1; Clark (n 54) 346.

129 United Nations Verification Mission in Colombia, Report of the Secretary General, S/2022/267 (28 December 2021 to 25 March 2022) <https://colombia.unmissions.org/sites/default/files/04.04.22_eng_infographic_reportmar2022.pdf> accessed 4 April 2022.

130 Colombian Constitutional Court (CCC), Sentence C-080 of 2018, 762.

131 ibid 747.

132 ibid 747, 748; Acto legislativo No 01 de 2017, Artículo transitorio 1^o^. For the CCC, the special sanctions linked to the aim of ending the situation of social exclusion have a reparative effect in a collective dimension because they seek to re-establish the collective rights of those affected communities and are *per se* a guarantee of non-repetition by responding to the structural factors causing violence.

133 ibid 288–91.

134 Acto legislativo No 01, Artículos transitorios 1^o^ and 5^o^.

135 Ley No 1957, arts 132, 140.

136 Special Jurisdiction for Peace, Sala de Reconocimiento de Verdad, de Responsabilidad y de Determinación de los Hechos y Conductas, Autos No 19 del 26 de enero 2021, 244 del 29 octubre 2021,125 del 2 de julio 2021, 128 del 7 de julio 2021.

137 Comisión Colombiana de Juristas, Boletín #53 del Observatorio sobre la JEP (14 febrero 2022) <www.coljuristas.org/observatorio_jep/documentos/documento.php?id=237> accessed 3 March 2022.

138 Colombian Unit for the Attention and Integral Reparations for Victims (n 2).

139 Misión de Verificación de las Naciones Unidas en Colombia, Informe del Secretario General, S/2022/267 (28 March 2022) <https://colombia.unmissions.org/sites/default/files/n2229476.pdf> accessed 4 April 2022.

140 ‘Derechos de las víctimas de las farc no se garantizan con la JEP: Herbin Hoyos’ *El País.com.co* (Cali, 6 July 2017) <www.elpais.com.co/proceso-de-paz/derechos-de-las-victimas-de-las-farc-no-se-garantizan-con-la-jep-herbin-hoyos.html> accessed 15 May 2021; ‘Duque pide sanciones “efectivas” para miembros de antiguas Farc’ *El Tiempo* (Bogotá, 28 January 2021) <https://www.eltiempo.com/politica/gobierno/duque-pide-sanciones-efectivas-para-miembros-de-antiguas-farc-563301> accessed 15 May 2021.

141 Ley No 1957, art 4.

142 SJP, *Manual para la participación de las víctimas ante la Jurisdicción Especial para la Paz* (Bogotá 2020) 34.

143 Enith Bula, Juan Carlos Ospina and Julián González, *Superando el dilema de la participación: El Funcionamiento de la JEP desde un enfoque basado en los derechos de las víctimas* (Comisión Colombiana de Juristas 2020) <www.coljuristas.org/documentos/libros_e_informes/Superando_el_dilema_de_la_participacion.pdf> accessed 27 June 2021, 69.

144 SJP (n 142) 134–48.

145 ibid 167–8; Ley No 1957, art 141.

146 Ley No 1922 de 2018, art 1.C.

147 The SJP has seven macro-cases, with hundreds or thousands of victims <https://www.jep.gov.co/especiales1/macrocasos/index.html> accessed 15 May 2021.

148 Anna Myriam Roccatello and Gabriel Rojas, ‘A Mixed Approach to International Crimes; The Retributive and Restorative Justice Procedures of Colombia’s Special Jurisdiction for Peace’ (ICTJ Report 2020) <www.ictj.org/publication/mixed-approach-international-crimes-retributive-and-restorative-justice-procedures> accessed 23 May 2021, 32.

149 CCC, Sentence C-080 of 2018, 265–6 and 762.

150 Currently, debates are taking place by practitioners and academics about the notions ‘determinative participation’ and ‘most responsible’, their possible interpretations and application. It is beyond the scope of the article to address this issue further. See Michalowski, Cruz and Martínez (n 25).

151 ICC OTP, Report on Preliminary Examination Activities (4 December 2017) paras 144–8; John Vervaele, ‘Transitional Criminal Justice in Colombia and Complementarity Policy under the Rome Statute of the International Criminal Court’ in Ulrich Sieber and others (eds), *Alternative Systems of Crime Control: National, Transnational and International Dimensions* (Duncker & Humblot 2018) 140.

152 ibid.

153 CCC, Sentence C-080 of 2018, 348.

154 Murphy (n 41) 162.

## Notes

This research was funded by The Netherlands Organization for Scientific Research (NWO), Grant Number 406-15-255. The authors would like to thank the anonymous reviewers for their constructive comments and valuable feedback.

